# Consideration of Some Matters Pertaining to Occlusion and the Occlusal Performance

**Published:** 1922-12

**Authors:** James Kendall Burgess


### CONSIDERATION OF SOME MATTERS PERTAINING TO OCCLUSION AND THE OCCLUSAL PERFORMANCE.

BY JAMES KENDALL BURGESS, D.D.S.

There is no more fascinating or vital study pertaining to the oral cavity than those matters that have to do with the relations of the teeth. What wonderful harmony runs through the plans of the Creator! The great apostle said, "And now abideth faith, hope, charity, these three; but the greatest of these is charity." The designation of charity as the most excellent of this great triumvirate of virtues is because at this point man comes to the parting of the ways, digresses from self and self-interest, and takes upon himself the relations, with their responsibilities, that his Creator designed he should bear to his fellow-creatures and finds his own greatest good and fullest happiness in a reaction from the service which these responsibilities entail. Applying this principle of the Divine purpose to the human denture we discover in its component parts dimension, conformation and relation, these three, but the greatest of these is relation, because, as complex and as wonderful as are the individual organs in conformation and color and

Read before The National Dental Association, Boston, Mass., August 23-27, 1920, printed in "Dental Cosmos," June, 1922.

in the variety and functions of the materials of which they are constituted, it is not as individuals—tooth for tooth—that they render their greatest service and gain their greatest measure of protection or prophylaxis, but it is in their relations to their fellows and their antagonists that their functions are performed, service rendered and prophylaxis fostered.

At this point two pictures present themselves to view. For the sake of dramatic effect we might label them "Before" and "After," and exclaim, "Look on this picture and then on that." The first is a human denture just come into the fulness of bloom, as it were— its development complete in all of its glory and harmony of form, color, arrangement and stability and in its wonders of mechanical details and relations, and unspoiled by accident, abuse or pathological development. The other is its very antithesis, a human denture, worn, disheveled, disorganized, its foundation in a state of dissolution, its units out of relation to their fellows and their antagonists and in varying stages of instability, being drawn hither and thither by every attempt at service—a picture of desolation and despair. The contrast between these two familiar pictures is appalling and one is led to consider why such a state as that represented by the second picture should ever exist. What are the causes that produce it or admit of its production and the stages by which it is developed? Certain it is that nature has not planned or desired it to be so.

She has made provision for the maintenance and the perpetuation of all her organs and tissues. It is inconceivable that a matter of such vital concern to the welfare of all the others as her dental equipment should alone be the object of her neglect in this regard, and a study of the human dental apparatus anatomically, histologically, physiologically and mechanically will readily convince us that it is not true. It is evident therefore that this tragedy is the result of some interference with her plans, aided and abetted by the operation of her immutable laws of cause and effect, of action and reaction, of force and resistance Let us consider then at least one factor—to your essayist the most vital factor—in its etiology and progress. The engineer who designs a great bridge or a high tower calculates the stresses to which his structure will be subjected through the play of natural forces and though the service for which it is constructed, adds the proper percentage for the factor of safety and builds his structure to meet these demands. Nature is not less wise.

To substantiate this declaration I beg to refer to a former essay entitled ''Periodontia as an Engineering Problem,'' read before the American Academy of Periodontology at its annual meeting in New Orleans, October 18, 1919, and published in the *Dental Items of Interest* for June, 1920.

In the article referred to we have outlined briefly the true nature of the dental apparatus, its character as a piece of mechanism designed for a specific achievement, its *modus operandi,* something of its component units or implements, and analyzed the passive and active relations which we speak of as ''occlusion'' and ''the occlusal performance'' respectively. Mention is made of the occlusal surfaces as the working parts of the teeth, and in an earlier descriptive paragraph ''the cuspated conformation'' and ''intercuspated relations'' of the antagonizing surfaces were spoken of.

This general description, however, is not sufficient for a complete understanding. A more detailed analysis will reveal a deep-laid plan in these conformations and relations and a corresponding penalty for its violation. These cusps, so called, are pointed and taper back from the points to broad bases at such angles as to provide heavy reinforcements and form planes of such shape, imposed at such angles and bearing such relations to similar planes of antagonizing cusps, as to constitute, when taken in series as they occur, a crushing, incising and triturating apparatus of wonderful efficiency. They are so shaped and have their points of attack and planes of service superimposed over the anchoring and supporting bases or roots in such positions and relations as to give them the greatest possible power and efficiency in the drive, the impact and the excursion, with the least possible strain upon the anchoring and supporting mechanism through any and all of these factors of the occlusal operation.

Here we have nature's normal in conformations and relations, or normal occlusion, producing her normal in service and function. We have her normal of stress or force generated in the occlusal performance acting against her normal of resistance inherent in the foundation, and creating her normal of reaction or physiological reflex in the foundation or supporting tissues. In other words, a situation in which nature has created her own balance between the resistance which she has provided in the foundation and the stress which she has designed should be brought against it in service. This constitutes function. Less than this is proportionately idleness or function minus and more is strain or function plus.

Nature's inexorable law of compensation is: Stimulus for service, measure for measure. This is simply a concrete statement of the fact that the reaction or reflex stimulation in the foundation tissues is always commensurate with the stress produced in the service of mastication. It is through this balanced service or function, then, that her organs and tissues are brought within their proper relations to the operator of this law of compensation, so that new materials, vitalized and energized, perpetually replace those consumed in function. Thus prophylaxis is maintained; and the only true prophylaxis is attained and maintained through function, both idleness and strain defeating its achievement through stagnation or overstimulation, as the case may be.

Prophylaxis as we use it in this connection is an inclusive term and comprehends not only the foundation of investigation and supporting tissues and the teeth individually, but all of the relations of tooth with tooth and tooth with investing tissues, including the cuspated conformations and intercuspated relations of the occlusal surfaces. There is no doubt in my own mind that nature planned so important a part of her greatest achievement, the human organism, to be self-sustaining, self-cleaning and self-correcting. That is to say that in nature's plan the physiological reflexes in the investment tissues from the mechanical functioning of the teeth should keep the foundation nourished and healthy; that the conformations of the teeth and the relations of the teeth and their investment tissues should preclude accumulations which necessitate external or supplemental aid or means in cleansing; and that the character and the relations of the antagonizing surfaces and the workings of the mechanism which controls their movements and their relations during function should so regulate the wear to which they are inevitably subjected as to conserve their cuspated conformations and intercuspated relations. It is probable that of all her organs and tissues the teeth alone are not compensated by the renewal or replacement of worn tissue, but a study of the relations of teeth formed, arranged, anchored and antagonized according to nature's plan will reveal that while wear is inevitable through the service for which they are designed, when all the details of her plan are adhered to there results a minimum of wear, and not only so but the wear is on such surfaces and planes and at such angles that the cuspated conformations and intercuspated relations are maintained and perpetuated.

A study of dentures and dentists, including myself, lrmly convinces me that the dentist was not in nature's plan but, like the deterioration which necessitates him, he is the product of the perversion of her purposes. But in order that nature may achieve this wonderful purpose of maintaining and perpetuating her apparatus through service or function it is necessary that her perfectly coordinated plan, in which all the parts are designed to fit one another like the cogs of a well-balanced piece of mechanism, shall be perfectly executed. Size, conformation, relations of all the parts, character and quality of tooth tissues and of foundation tissues, and metabolism, each must play its part. A casual observation, however, will reveal that in many instances nature has been thwarted of her purpose; disturbing elements have injected discord into her plans with resulting disharmony in their execution and a greater or less degree of havoc as the ultimate product.

To enumerate these disturbing elements would be to give a list of all dental and oral defects and deficiencies and abnormalities to which the flesh is heir, every one of which alters nature's perfect balance and holds possibilities for evil far outweighing the measure of its own deviation from the normal. They are matters of everyday observation to the general practitioner as well as to the specialist. These variations from the normal may be classified as congenital and acquired.

Of the congenital defects the more conspicuous perhaps and those that concern us here more particularly are deviation from type—generally manifested in inadequacy of development as to cusp and contour—and lack of balance or correlation between the sizes of the teeth and their containers or jaws. This latter defect is manifested on the one hand in what we might term a minus relation, by which I mean a condition in which teeth of subnormal size occupy jaws of normal size or teeth of normal size, jaws of supernormal size; on the other, by a plus relation, in which teeth of normal size occupy a subnormal or underdeveloped jaw or teeth of supernormal size occupy a jaw otherwise relatively normal. These conditions taken in connection with the deviation from type or conformation so frequently associated with the minus relations preclude normal relations both proximal and occlusal; as do also conditions due to the loss of proximal relations and support through caries, extraction or operative inefficiency.

Herein lies the great importance of correct relative sizes of teeth and jaws because without this there can not be correct proximal relations, and without correct proximal relations there can not be correct occlusal relations. A tooth that occludes, with a space at the contact point the thickness of a sheet of paper, is out of occlusion just as surely as though it occluded with the space from which a tooth has been extracted. The principle is the same and the difference one only of degree.

Nature's occlusion, it matters not whether we speak of it as perfect or ideal or normal or what not, is not a mere matter of external appearance but of inward exactness of relations. It is part of a comprehensive scheme which necessitates exactness in conformation, alignment, anchorage, proximal relations and every other detail that nature planned for it.

Nature's plan for perfect occlusion thus having not materialized or her perfect relations being disturbed, there results attrition which produces not only a progressive change in form and relations of both cusp and plane, but a progressive shifting of the points of attack and the planes of service from the normal or natural positions in their relation to the supporting base or root.

Attention has been called to the significance of this condition, from the engineering point of view especially, by Dr. Isador Hirschfeld of New York City. The extreme of this condition is the case where the cusps are entirely lost and instead of a series of cusps we have the broad flat horizontal surface or plateau of the dimensions of the largest circumference and greatest diameter of the tooth. The man in the street can readily understand the difference in the amount of force required and the resulting stress in driving teeth with cuspated occlusal surfaces in their intercuspated relations, and those where abrasion has left broad horizontal planes through the bolus of food.

The inference here is very plain. Teeth in the relations which include all the elements of nature's own plan for the occlusion not only exhibit this cuspated conformation in intercuspated relations but in the performance of the service which nature intended them, are constantly being whetted or honed as it were against each other on such planes and at such angles as that the inevitable wear conserves, at least in principle, these conformations and relations. Teeth in other than nature's intended relations wear on different planes and at different angles, altering or destroying their cusps and presenting dulled, and ultimately flattened, surfaces to the food bolus, requiring greatly increased power in the attack and a large increase in the required number of attacks for a given result, with a commensurate increase in the stress produced against the foundation. Here we have in one case physiological wear brought about through physiological relations and function or service and conserving a physiological equilibrium, and in the other case pathological attrition resulting from abnormal relations, productive of strain or overloading and eventuating in disorganization—in one case a balanced occlusion according to nature's plan in which is developed a maximum of power and efficiency, producing normal stress with only such reaction in the foundation tissues as constitutes function and conserves in the greatest possible degree their anatomical and physiological integrity, and in the other case a plus or overloaded occlusion in which as we retrogress from nature's perfectly balanced plan we have a progressive decrease in power and efficiency, with progressively increasing stress and a corresponding reaction in the supporting tissues that becomes pathological instead of physiological and amounts ultimately to trauma, dissolution of the foundation and disorganization of the denture.

What is the remedy? So far as details of technique are concerned I confess I have not brought you one and I am well aware that this will make me *persona non grata* and even *non compos mentis,* as it were, with that element in the profession which insists at every turn upon some new method of constructing the shell crown, some new argument for or against the direct or the indirect method of inlay construction, or some new device that will add to the joys of the mechanical details of construction of the slip-on type of bridge restoration. Important as these things are they are not all of dentistry. If it be true, as there is no room to doubt, that "the iniquity of the fathers is visited upon the children unto the third and fourth generation," it would seem that our first recourse would be to go back these three or four generations and exercise greater care in choosing our ancestors, and if we have the good of humanity at heart, to take such steps as the deepest minds and the greatest hearts among us devoted to the most intelligent and sacrificing research can discover or devise for the overcoming of these defects and deficiencies for the unborn generations. But there are immediate problems to be solved here.

The result of all occlusal effort is force or stress, and stress necessitates a commensurate reciprocal force or resistance in the foundation. In the case of plus occlusion, whether due to an overloaded foundation or an undermined occlusion, the occlusal stress is out of proportion to the foundation resistance, and it is imperative that they shall be balanced. The difficulties of adding materially to the strength of the foundation are in many instances insurmountable, and where we can not establish an equal or a plus relation in the foundation we are left with the sole expedient of dealing with the occlusion. Since the occlusal operation is nature's established mechanical procedure which we are powerless to alter, we are reduced to the necessity of handing the three factors which enter into occlusion, *viz.*, the area, conformation and relation of the occlusal surfaces, either singly or in such combination as will best serve the purpose of reducing the plus in the relation of occlusion to foundation to a balanced or even a minus relation.

I have laid down here a principle of action. Combinations of conditions and relations are so numerous and methods of technical procedure which they admit or necessitate so varied that it is not expedient or wise to enter here on that phase of the subject. The imperative thing is that we come to a full realization that the teeth exist for service; that service is performed through the occlusal operation; that the burden of this performance falls on the foundation in which the teeth are anchored; that the occlusal surfaces and relations can not render more service than the foundation can produce; that occlusal relations which necessitate overloading to produce normal service are plus to a normal foundation; that occlusal relations designed for normal service are plus to an impaired or minus foundation; and that if teeth are going to be kept in their foundations and in service they must be kept in balanced relations.

It would seem that the lessons in all of this should be apparent to both orthodontist and periodontist. The orthodontist must know that there is much more to correct occlusion than can be seen with the teeth closed and the lips open, and the periodontist must understand that the best periodontic procedure comprehends as an outstanding feature correct orthodontic practic. The highest mission of the orthodontist must be prophylactic periodontia and the greatest achievement of the periodontist, prophylactic orthodontia. But because the specialists in either of these matters are so few compared with the general practitioners it is upon this latter class that the great burden and responsibility must fall, and to fulfil his greatest purpose the general practitioner must be both orthodontist and periodontist in this broad prophylactic sense that I have indicated.

This brings me to the assertion that the plane of occlusion is the sacred place—the holy ground as it were—of the oral cavity. It is not only the plane of duty and of service, which alone gives to every created thing the right to existence, but the plane of prophylaxis in the inclusive sense which comprises the conservation of the foundation and of the conformations and relations of the teeth that constitute the denture—so wonderful as an artistic creation, so marvelous as an engineering achievement, but so tragic in its desolation.

What modern Nehemiah will rise up to build again these walls of Jerusalem?

